# Epitaxial Growth of GaN Films on Chemical-Vapor-Deposited 2D MoS_2_ Layers by Plasma-Assisted Molecular Beam Epitaxy

**DOI:** 10.3390/nano14080732

**Published:** 2024-04-22

**Authors:** Iwan Susanto, Hong-Shan Liu, Yen-Ten Ho, Ing-Song Yu

**Affiliations:** 1 Department of Materials Science and Engineering, National Dong Hwa University, Hualien 974301, Taiwan; iwan.susanto@mesin.pnj.ac.id (I.S.);; 2 Department of Mechanical Engineering, Politeknik Negeri Jakarta, Depok 16424, Indonesia; 3 International College of Semiconductor Technology, National Yang Ming Chiao Tung University, Hsinchu 300093, Taiwan; chia500@yahoo.com.tw

**Keywords:** gallium nitride, molybdenum disulfide, molecule beam epitaxy, chemical vapor deposition, van der Waals epitaxy

## Abstract

The van der Waals epitaxy of wafer-scale GaN on 2D MoS_2_ and the integration of GaN/MoS_2_ heterostructures were investigated in this report. GaN films have been successfully grown on 2D MoS_2_ layers using three different Ga fluxes via a plasma-assisted molecular beam epitaxy (PA-MBE) system. The substrate for the growth was a few-layer 2D MoS_2_ deposited on sapphire using chemical vapor deposition (CVD). Three different Ga fluxes were provided by the gallium source of the K-cell at temperatures of 825, 875, and 925 °C, respectively. After the growth, RHEED, HR-XRD, and TEM were conducted to study the crystal structure of GaN films. The surface morphology was obtained using FE-SEM and AFM. Chemical composition was confirmed by XPS and EDS. Raman and PL spectra were carried out to investigate the optical properties of GaN films. According to the characterizations of GaN films, the van der Waals epitaxial growth mechanism of GaN films changed from 3D to 2D with the increase in Ga flux, provided by higher temperatures of the K-cell. GaN films grown at 750 °C for 3 h with a K-cell temperature of 925 °C demonstrated the greatest crystal quality, chemical composition, and optical properties. The heterostructure of 3D GaN on 2D MoS_2_ was integrated successfully using the low-temperature PA-MBE technique, which could be applied to novel electronics and optoelectronics.

## 1. Introduction

Gallium nitride (GaN), a group III-V compound semiconductor material, has a strong bonding force, a direct energy gap, good thermal conductivity, and excellent radiation resistance [1]. Therefore, it has been utilized in many electronic and optoelectronic devices, such as laser diodes, light-emitting diodes [2], high-speed electronic transistors [3], UV photodetectors [4], solar cells [5], photocatalysts for water splitting [6], and more. However, the epitaxial growth of high-quality GaN films for these applications is still limited by the mismatches in lattice parameters, thermal expansion coefficients, and process parameters for different epitaxial technologies [7]. In conventional epitaxy, suitable substrates or proper buffer layers in accordance with the lattice constant of GaN are in high demand to support further applications of GaN devices. In addition, an interesting research field concerning 2D transition metal dichalcogenides (TMDs) has recently become very popular due to its great potential in nanoelectronics applications [8]. Molybdenum disulfide (MoS_2_), one of the 2D TMD materials, has excellent optical, electrical, and physical properties [9]. This material has the characteristics of transferability due to the van der Waals bonding between layers. Additionally, the weak van der Waals interactions and lack of dangling bonds on TMD surfaces might help to manage stress between the epitaxial layer and substrate, known as van der Waals epitaxy. It means the 2D material completely screens the substrate potential and substitutes the role of the substrate such that the epitaxial layer nucleates and grows directly on the 2D material rather than the underlying substrate [10]. Therefore, van der Waals (vdW) epitaxy on two-dimensional (2D) materials has opened a new opportunity for epitaxial growth, overcoming the material’s compatibility issue [11], which also could provide a freestanding and high-quality nanomembrane for massive novel applications [12]. The epitaxial growth of thin films on 2D materials can subsequently be transferred to different substrates for further applications [13].

Moreover, the integration of two-dimensional MoS_2_ with GaN recently attracted significant attention for future electronic or optoelectronic applications. Giannazzo et al. reported the growth of uniform MoS_2_ films on homoepitaxial n-GaN substrates, characterized by low tensile strain and significant p^+^-doping. The demonstration of a nearly ideal van der Waals interface between MoS_2_ and GaN highlighted the potential for integrating these materials to advance vertical heterojunction diodes, which held promise for high-power and high-frequency device applications [14]. Moreover, various methods have been proposed to create the heterojunctions of 2D MoS_2_ and 3D GaN materials. These approaches included extracting MoS_2_ flakes from bulk crystals [15], transferring MoS_2_ thin films grown on different substrates [16], and directly growing MoS_2_ on GaN substrates using scalable techniques such as chemical vapor deposition (CVD) [17] and pulsed laser deposition (PLD) [18].

Molecular beam epitaxy (MBE) is a relatively low-temperature process, so the integration of heterojunctions provides a pathway for developing GaN/MoS_2_ film heterostructures, with 2D MoS_2_ materials serving as substrates or buffer layers [19]. One more interesting thing is that MoS_2_ has an extremely small lattice mismatch (0.9%) with GaN for reducing the residual strain and increasing the crystal quality of GaN films on the hetero-layers [20]. The study demonstrated that single-crystal islands of GaN have been grown on mechanically exfoliated flakes of MoS_2_ [21]. However, detailed studies on GaN epitaxial growth on large-area and single-crystal 2D MoS_2_ layers have not shown promising results. Susanto et al. reported the characterization of ultra-thin GaN films grown on different 2D MoS_2_ layers prepared by PLD and CVD, which indicated that the quality of 2D MoS_2_ can influence the epitaxial growth of GaN [22]. It is noted that there is still a challenge for growing high-quality GaN on 2D MoS_2_ layers. An advanced investigation of GaN/MoS_2_ heterostructures by MBE was proposed for different parameters of pre-nitridation, growth temperature, and differing growth durations [23]. Recently, the growth of large-area and high-quality 2D MoS_2_ was realized using the CVD technique [24]. It also means a higher possibility of GaN/MoS_2_ heterostructures for future electronic and optoelectronic devices.

Several studies have attempted to enhance the quality of GaN films by adjusting the flux ratio between N and Ga atoms [25]. Tsai et al. reported that the reduction in defects was related to mixed-type dislocations that occurred in GaN films grown on the sapphire at 788 °C as the N/Ga flux ratio increased [26]. Susanto et al. investigated whether the crystal quality of GaN grown on a SiC substrate can be improved by constant N flux and increasing Ga flux since the flux ratio of N and Ga atoms has decreased to near Ga-rich growth conditions [27]. However, the suitable Ga flux for the optimization of GaN growth still has challenges for a different kind of substrate or buffer layer. The properties of substrates including thermal expansion coefficients, thermal conductivities, lattice constants, defect densities, elastic moduli, and etching characteristics strongly influenced the growth mechanism of epitaxial layers [7]. Therefore, an investigation into the epitaxial growth of GaN films on 2D MoS_2_ layers with the appropriate parameters is exhaustively needed to resolve the above issue.

In this work, GaN thin films have been epitaxially grown on two-inch wafer-scale 2D MoS_2_ layers y using low-temperature plasma-assisted molecular beam epitaxy (PA-MBE) in order to fabricate the 3D GaN and 2D MoS_2_ heterostructures. The few-layer MoS_2_ was prepared using the CVD technique and played the role of substrate for GaN growth. In MBE growth, we investigated variable sources of Ga flux provided by a Knudsen effusion cell (K-cell) and the constant N flux by an RF plasma source. Moreover, the characterization of GaN thin films was conducted using the observations of surface morphology, chemical composition, crystalline structure, and optical properties. This work not only can illuminate critical issues in the epitaxial growth of conventional semiconductors on 2D crystals for various device applications but also can demonstrate van der Waals epitaxial growth of large-area and high-quality GaN films on 2D MoS_2_.

## 2. Materials and Methods

For the preparation of substrates, 2D MoS_2_ layers were deposited on a 2-inch c-sapphire substrate and the growth of the layers was carried out in the CVD system using a reaction between H_2_S gas and MoO_3_. Two steps of the growth technique were performed in the layer construction process. First, the ultra-thin layer of MoO_3_ (around 1 nm) was grown on the sapphire substrate from powder with a high purity of 99.95% and a high vacuum E-gun evaporator. Second, a sulfurization process using H_2_S (10%) gas was conducted at a temperature of 750 °C to synthesize the high-quality 2D MoS_2_ layers in the furnace. This chemical-vapor-deposited 2D MoS_2_ layer on sapphire played the role of the substrate for the growth of GaN films. The growth of the 2D MoS_2_ layers was carried out using a reaction between H_2_S gas and MoO_3_, which can be represented by the following chemical equation [28]:

3H_2_S_(g)_ + MoO_3(s)_ → 3H_2_O_(g)_ + MoS_2(s)_ + S_(s)_ (1)

For the epitaxial growth of GaN using a ULVAC PA-MBE system, the parameters of epitaxial growth are listed in Table 1. The samples A*, B*, and C* were noted as GaN films grown at gallium (Ga) K-cell at the temperatures of 825 °C, 875 °C, and 925 °C, respectively. Before the GaN films were grown, 2D MoS_2_ layers were characterized using Atomic Force Microscopy (AFM), Field-Emission Scanning Electron Microscopy (FE-SEM), and Raman Spectroscopy. During the growth of GaN films, the surface structure was monitored using in situ Reflection High-Energy Electron Diffraction (RHEED). The base pressure of the MBE chamber was maintained in an ultra-high vacuum of 5 × 10^−8^ Pa. Further, the thermal cleaning and pre-nitridation treatment of 2D MoS_2_ layers were carried out at 600 °C for 30 min and 10 min, respectively. Then, GaN films were grown at the substrate temperature of 750 °C for 3 h with different Ga fluxes, generated from various K-cell temperatures. Meanwhile, a nitrogen plasma source was provided by an N_2_ flux of 0.8 sccm and 500 W RF power.

After the growth of GaN films, all samples were carried out with ex situ characterization. Surface roughness and morphology were observed using JEOL JSM-7000F FE-SEM and Nano Surf C3000 AFM, respectively, while the vibration modes of GaN and MoS_2_ were identified using room-temperature Raman spectroscopy equipped with a 532 nm laser. The optical properties of GaN films were investigated using room-temperature photoluminescence (PL) using a 266 nm UV laser. Surface chemical composition was examined using VGS Thermo K-Alpha X-ray photoelectron spectroscopy (XPS). Thus, the crystallography was investigated using high-resolution X-ray Diffraction (HR-XRD) and Transmission Electron Microscopy (TEM), JEOL JEM-2100F. Finally, the element composition of the GaN/MoS_2_ heterostructure was identified using Energy-dispersive X-ray spectroscopy (EDS).

## 3. Results and Discussion

### 3.1. RHEED

**Figure 1:**
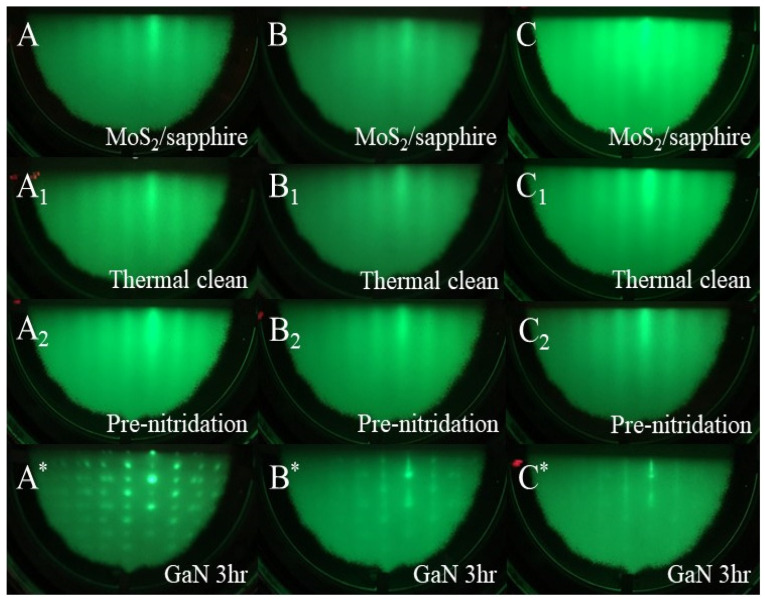
RHEED patterns: MoS_2_/sapphire (**A**, **B** and **C**) before thermal cleaning, MoS_2_/sapphire (**A_1_**, **B_1_** and **C_1_**) after thermal cleaning, MoS_2_/sapphire (**A_2_**, **B_2_** and **C_2_**) after pre-nitridation, and GaN films on MoS_2_/sapphire after the growth with gallium K-cell at 825 °C (A*), 875 °C (B*), and 925 °C (C*).

Figure 1 shows RHEED patterns of three samples during the epitaxial growth of GaN films. First, the images of A, B, and C are the RHEED patterns of the substrates with 2D MoS_2_/sapphire prepared by CVD. Second, A_1_, B_1_, and C_1_ are the patterns after the thermal cleaning process. Then, A_2_, B_2_, and C_2_ are the patterns after the pre-nitridation process. Finally, A*, B*, and C* are the patterns of GaN films grown on the substrates. For the RHEED images of 2D MoS_2_/sapphire substrates, all patterns displayed streaks. The streaky patterns correspond with both the flat surface and the crystal structure with small domains for CVD MoS_2_ layers, which also indicate the same surface condition of MoS_2_ layers for the following epitaxial growth of GaN thin films.

After the growth of GaN films on 2D MoS_2_/sapphire (A*, B*, and C*), the RHEED patterns provided crucial information about the quality and morphology of the deposited layers. At a Ga K-Cell temperature of 825 °C, RHEED pattern A* exhibits bright spot patterns, indicating the formation of single-crystal wurtzite GaN films on the substrate [29]. Additionally, the presence of spot patterns suggests a rough surface morphology with three-dimensional islands, characteristic of 3D growth mechanisms [26,30]. When the Ga K-Cell temperature increases to 875 °C, as seen in RHEED image B*, streaky patterns coincide with the spot configurations, which implies a transition in the growth mechanism of GaN. Finally, at a Ga K-Cell temperature of 925 °C, RHEED pattern C* shows a flat and crystalline GaN surface with multiple terraces, indicative of the 2D growth of GaN. The additional Ga flux at this higher temperature appears to stimulate more organized growth, yielding a flatter surface with more terraces, both of which are signs of improved epitaxial growth.

### 3.2. SEM

**Figure 2:**
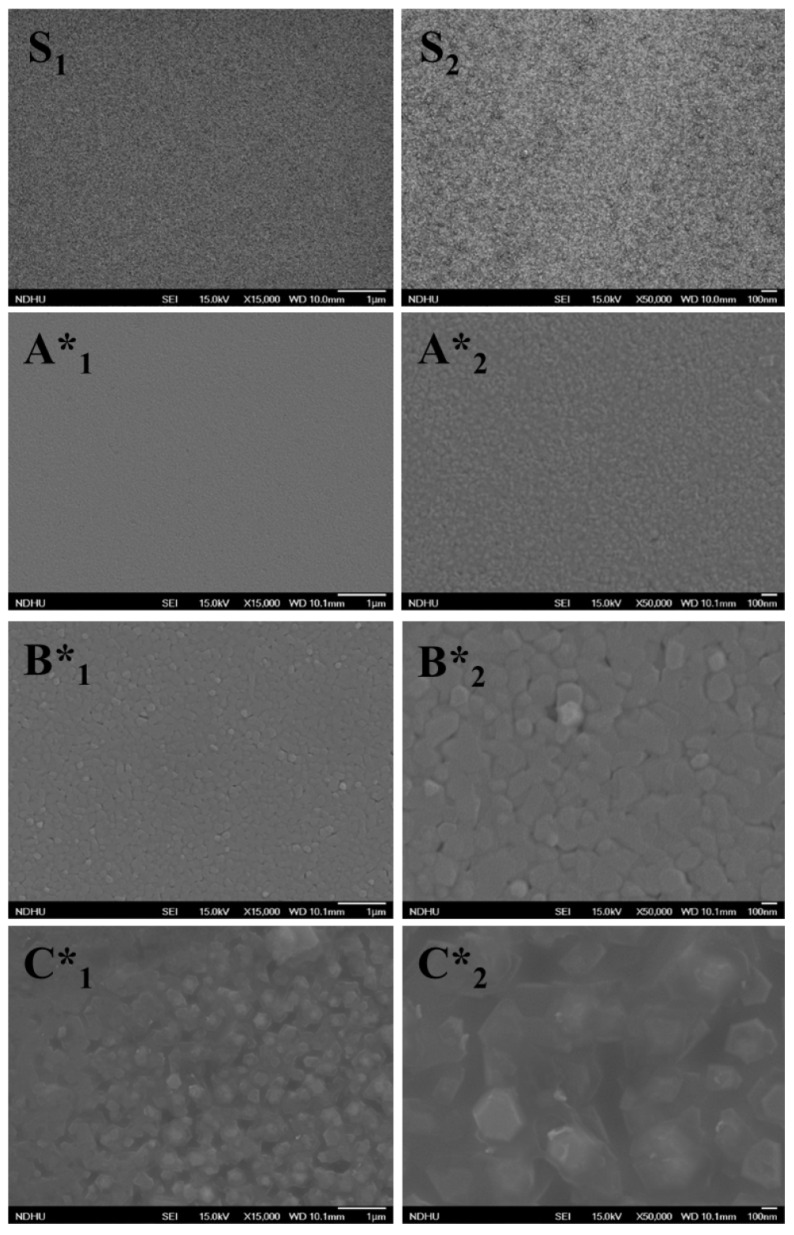
FE-SEM images of 2D MoS_2_ layers/sapphire (**S_1_** and **S_2_**) and GaN/2D MoS_2_/sapphire at the magnifications of 15,000× (A*_1_, B*_1_ and C*_1_), and 50,000× (A*_2_, B*_2_ and C*_2_).

Figure 2 shows FE-SEM images for the surface morphology of the substrate (S) and three samples of GaN films (A*, B*, and C*), which are denoted as 1 and 2 for different magnifications. Images S_1_ and S_2_ display the homogeneous morphology of CVD MoS_2_ layers, but it is possible to observe some small particles in image S_2_. For sample A*, the surface morphology of GaN films with a higher magnification, image A*_2_, displays small grains spread evenly on the surface. The morphology of GaN appears homogeneous on the 2D MoS_2_/sapphire substrate. With the increase in Ga flux during epitaxial growth, the morphology of sample B* (images B*_1_ and B*_2_) displays larger grains compared to sample A*. In sample C*, the images clearly exhibit wurtzite grains with an average size of 200 nm. This suggests that the sufficiency of Ga flux provided by a higher Ga K-cell temperature could facilitate the growth of wurtzite and larger grains of GaN films. In addition, the hexagonal and large grains indicate terraces with a multilevel surface, which is consistent with the observation of RHEED patterns.

### 3.3. AFM

**Figure 3:**
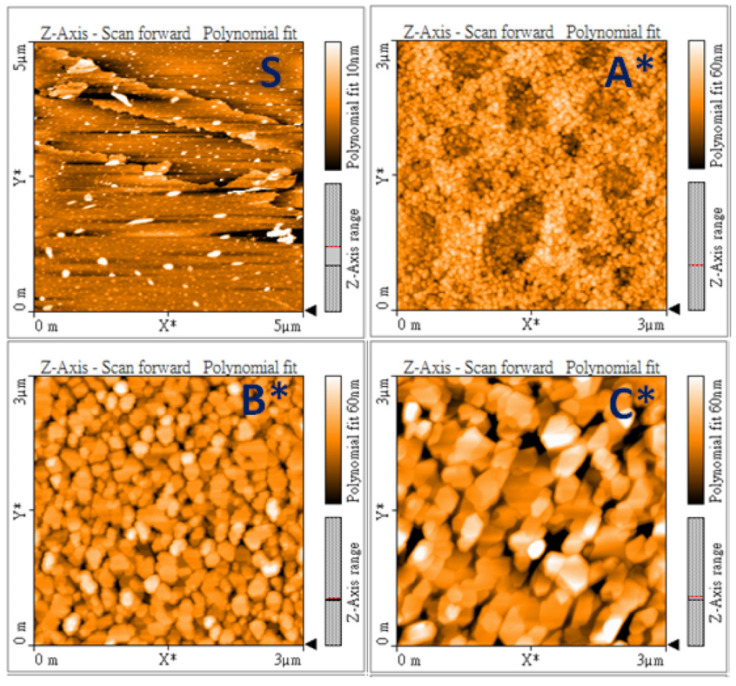
AFM images of surface 2D MoS_2_ layers/sapphire (**S**) and GaN/2D MoS_2_/sapphire (A*, B* and C*).

The surface morphology of MoS_2_ layers and GaN films can also be examined using the AFM technique. Figure 3 demonstrates AFM images in the scanning area of 3 × 3 μm^2^, including MoS_2_ (S) and three GaN films (A*, B*, and C*). The few-layer 2D MoS_2_ on c-sapphire by CVD is smooth for the growth of GaN, but some small particles are observed on the surface. After the growth, the surfaces of three GaN films display granular structures. Higher Ga flux is supplied during the MBE growth, and larger grain sizes can be observed on the surface of GaN films. We also note that larger terraces of GaN films via the mechanism of 2D epitaxial growth can be conducted at higher temperatures of the Ga K-cell.

### 3.4. XPS

**Figure 4:**
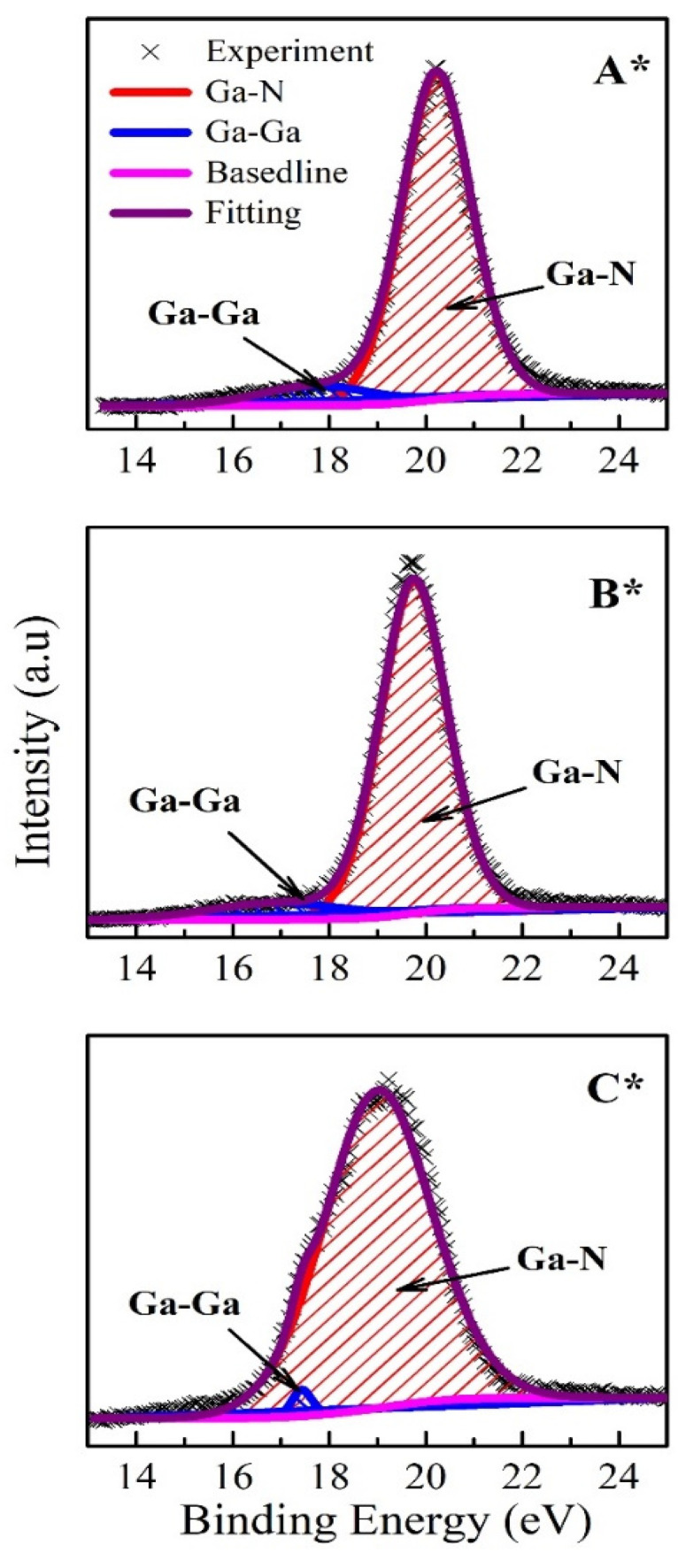
Deconvolution of Ga-3d XPS spectra for three GaN films on 2D MoS_2_/sapphire (A*, B* and C*).

The surface chemical composition of GaN films can be investigated using XPS spectra. Figure 4 displays the deconvoluted spectra of Ga-3d for three GaN films (A*, B*, and C*). The Ga-3d XPS spectra are divided into two typical regions of Ga-Ga and Ga-N bonding. Based on XPS fitting using *Avantage* software, the peak positions and bonding percentages of elements are shown in Table 2. The peak positions of Ga-N bonding in the orbital area of Ga-3d are located at 20.1 eV, 19.7 eV, and 19.1 eV for samples A*, B*, and C*. Meanwhile, those of Ga-Ga bonding are in the positions of 17.8 eV, 17.9 eV, and 17.4 eV, respectively. The shift towards a higher BE of 0.4 eV for the Ga-N peak position (sample A*) reveals an amount of surface oxidation, which is initiated by atmospheric exposure to samples [31]. Meanwhile, the opposite shift to a lower BE of 0.6 eV (sample C*) is related to the number of Ga metallics present on the GaN films [30]. Moreover, the percentages of Ga-N bonding are obtained at 90.9%, 90.6%, and 98.4% for A*, B*, and C*, respectively. Sample C* has the highest percentage of Ga-N bonding, which demonstrates the stable formation of the GaN film in growth with a high Ga flux condition.

### 3.5. Raman

**Figure 5:**
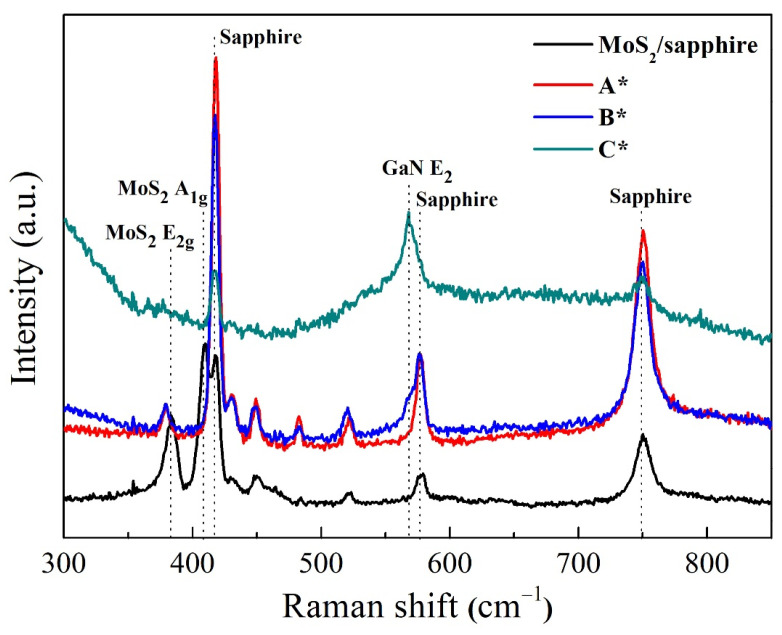
Raman spectra of substrate (MoS_2_/sapphire) and three GaN/2D MoS_2_/sapphire (A*, B* and C*).

The specific molecular bond vibration of the substrate and films can be identified using Raman spectroscopy at room temperature. This technique is used to study vibrational, rotational, and other low-frequency modes in a material by observing how incident light scatters off it. Figure 5 represents the measured results of 2D MoS_2_ layers and three GaN films (A*, B*, and C*). The peaks of 382 cm^−1^ and 408 cm^−1^ in the black curve (MoS_2_/sapphire) correspond to the characteristic MoS_2_ vibration modes of E_2g_ and A_1g_ phonons [32,33]. E_2g_ is associated with the displacement of sulfur and molybdenum atoms in the basal plane. A_1g_ corresponds to the atomic vibration of sulfur upright to the basal plane. After the growth of GaN films, the intensity of these two peaks becomes weaker, particularly in sample C*. The peak position of 567.9 cm^−1^ in the Raman spectra can be observed gradually, corresponding to the E_2_ vibration mode of GaN [32].

Moreover, the E_2_ phonon is the most sensitive in recognizing the attending stress in GaN films by calculating the shift mode. Generally, the up-shift and down-shift of the E_2_ phonon mode correspond to the compression and tensile strain compared to the strain-free GaN at 567.8 cm^−1^ [34]. The residual stress can be quantified using the following equation [13]:

σ = Δω/4.2 GPa(2)

where σ is in-plane stress and Δω relates to the experimental Raman shift mode from the peak position of stress-free. In-plane stress refers to the stress that acts parallel to the surface of the material, affecting its mechanical properties. This represents the stress within the plane of the GaN film. Meanwhile, Δω represents the change in the Raman shift of a specific phonon mode, denoted as E_2_, due to the presence of stress in the GaN film. In this case, the change in the frequency of the E_2_ phonon mode is indicative of the presence and magnitude of stress in the GaN film. Thus, according to the Raman spectra results shown in Figure 5, the residual stress of the GaN film, related to the up-shift peak mode of 0.1 cm^−1^, is calculated to be 0.024 GPa. The very small compressive stress confirms the release effect of internal stress from MoS_2_ layers, leading to the relaxation structure of the GaN film. This result could be generated because the GaN film is grown on the van der Waals bonding 2D MoS_2_ layer. van der Waals bonding, characterized by weak intermolecular forces, is prominent in 2D materials like MoS_2_. This bonding between individual atomic layers facilitates easy sliding or separation. When GaN films are grown on 2D MoS_2_ layers, the weak van der Waals bonding influences the growth dynamics, offering flexibility for GaN atom arrangement. This flexibility enables a stress-release mechanism during GaN deposition on MoS_2_, allowing the film to adjust more easily and partially relieve inherent stress or strain. It may also confirm the growth mechanism of van der Waals epitaxy for 3D GaN films on 2D MoS_2_ layers.

### 3.6. PL

**Figure 6:**
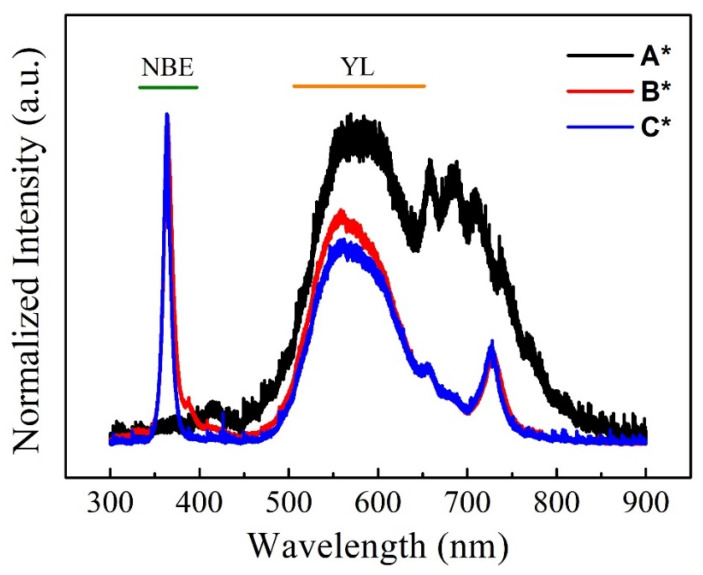
PL spectra at room temperature of three GaN/2D MoS_2_/sapphire (A*, B*, and C*).

**Figure 7:**
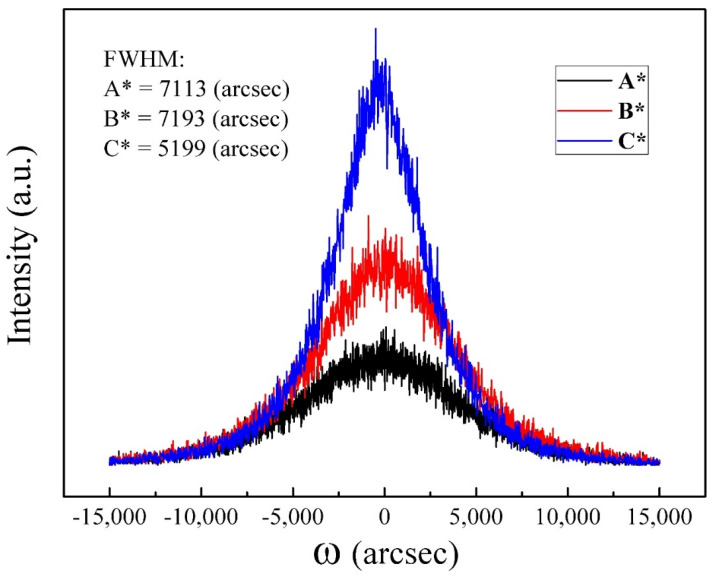
Rocking curve HR-XRD of GaN films on MoS_2_/sapphire in (0002) plane (A*, B*, and C*).

**Figure 8:**
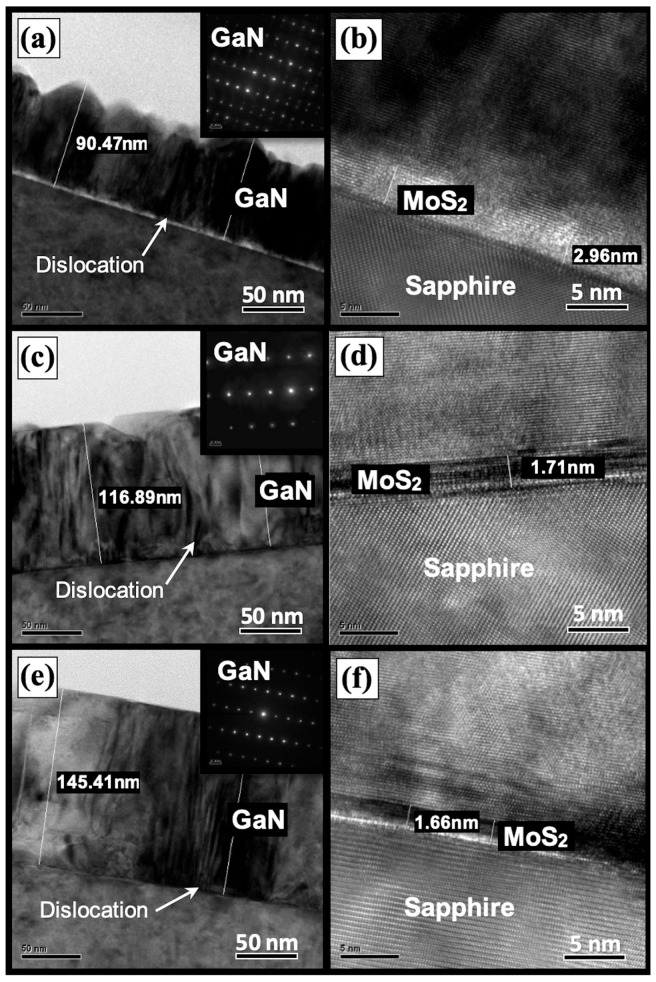
Cross-sectional TEM images of GaN films on the substrate 2D MoS_2_/sapphire: images (**a**,**b**) for sample A*, images (**c**,**d**) for sample B*, and images (**e**,**f**) for sample C*, respectively. Insets are SAD patterns of GaN films for three samples.

To clarify the optical properties of GaN films at distinct fluxes of Ga, PL spectroscopy can be employed to obtain a transition of electronic states. Figure 6 displays room-temperature PL spectra of GaN films, where the black, red, and blue curve colors correspond to samples A*, B*, and C*, respectively. The narrow and sharp peak, shown as the near band edge emission (NBE) of GaN films, is located at around 363 nm. Another broad peak, corresponding to yellow luminescence (YL), is between the wavelengths of 480 and 700 nm. The NBE peak is ascribed to the excited electrons in the radiative transition from the conduction band to the valence band. Thus, the YL peak reflects the defect state in GaN films [35]. For wurtzite bulk GaN, NBE peak emission is located at ~3.41 eV, and the YL peak is in the range of 1.4–2.8 eV [36]. In comparison with strain-free GaN films, the blue shifts of both 0.3 meV and 1.1 meV to higher energy were attributed to compressive stress in GaN films for samples B* and C*, respectively. Furthermore, in GaN films grown on 2D MoS_2_ layers with van der Waals bonding, the blue shift may also arise from inherent defect structures, notably dislocations, within the GaN films. Dislocation defects can affect the optical properties of the material by introducing new energy levels in the bandgap, potentially resulting in a blue shift in the emission or absorption spectrum. These defects have been confirmed by the broadening of peaks on HR-XRD in Figure 7, and further details are demonstrated by cross-sectional TEM images in Figure 8. As a result, the shifting of the NBE peak might produce residual stress of approximately 0.011 GPa for B* and 0.041 GPa for C*. It has been estimated that the shifting of the NBE peak by approximately ~27 meV, as well as 4.2 cm^−1^ from the Raman E_2_ phonon, may generate 1 GPa of biaxial strain [30]. The results of PL analysis show good agreement with the Raman spectrum. Meanwhile, the normalized intensity of the NBE peak displayed a sharp peak of both samples with full-width half-maximum (FWHM) of 120 meV and 86 meV. The lowest FWHM for sample C* reveals a higher quality of GaN film. On the other hand, sample A* shows a higher intensity of the YL peak, demonstrating a highly defected state constructed in GaN films. It indicates that the sufficiency of Ga flux could facilitate better optical properties of GaN films through both the improvement of the crystalline structure and a reduced defect state.

### 3.7. HR-XRD

To evaluate the crystal quality of GaN films, the measurement of HR-XRD has been carried out. Figure 7 shows the profile of the rocking curve in the (0002) plane of GaN films. Symmetric FWHM values are obtained as 7113 arcsecs for A*, 7193 arcsecs for B*, and 5199 arcsecs for C*. The values correspond to the quality of the crystalline structure [37]. Smaller FWHM values represent better crystal quality of films, while the broadening peak is associated with threading dislocations formed during the coalescence of GaN islands at the initial stages of epitaxial growth [38,39]. It is noticeable that GaN films grown with a higher Ga flux could suggest much better crystalline quality than those with a lower Ga flux. Based on the HR-XRD results from the rocking curve, sample C* has a lower defect density in the GaN crystal compared to samples A* and B*. The result is also consistent with the room-temperature PL spectra. The higher the crystallinity of GaN films, the better optical properties that can be performed in the PL measurements.

### 3.8. TEM

The heteroepitaxial structure of GaN films on 2D MoS_2_ can be analyzed using cross-sectional TEM observation. Figure 8 demonstrates three TEM images with the selective-area diffraction (SAD) measurements. In the low magnification shown in Figure 8a,c,e, the thickness of GaN films can be estimated as 90.47 nm for sample A*, 116.89 nm for sample B*, and 145.41 nm for sample C*. The higher the Ga flux we have during the epitaxial growth, the thicker GaN films we can obtain. Meanwhile, the inset images in Figure 8a,c,e show the SAD patterns of GaN films for the three samples. They also confirm that van der Waals epitaxial growth of GaN films has been realized successfully on 2D MoS_2_ layers. Moreover, the GaN surface morphology in the TEM images also confirms the growth mechanism from the 3D to 2D mode. The results are consistent with the analyses of in situ RHEED, SEM, and AFM. Finally, in the high magnification of high-resolution TEM images, displayed in Figure 8b,d,f, few-layer 2D MoS_2_ can be observed between sapphire and GaN films. The thickness of MoS_2_ layers is approximately 2.96 nm, 1.71 nm, and 1.66 nm for samples A*, B*, and C*, respectively. 2D MoS_2_ layers were not degraded during the low-temperature growth process by PA-MBE. We also observed evidence of the successful integration of GaN/MoS_2_ heterostructures using the MBE technique.

From the TEM observations, the selective-area diffraction pattern can confirm the single-crystal GaN films on CVD MoS_2_ via the van der Waals epitaxy. However, the defect related to dislocation is present in the films. The dislocations can be observed to have initiated from the interface between the MoS_2_ layer and the GaN film. This dislocation defect is thought to arise due to various factors. These factors include disparities in the crystal structure and differences in the thermal expansion coefficients between the two layers, as well as van der Waals interactions and the surface quality of MoS_2_ layers. As seen in the observations using SEM (Figure 2) and AFM (Figure 3), small particles on the MoS_2_ surface have been detected. These small particles can serve as nucleation sites for the formation of dislocation defects. They can act as starting points where dislocations begin to form due to disturbances in the atomic arrangement caused by the presence of these particles. During the epitaxial growth of GaN films, these particles may induce a 3D growth mechanism at the lower Ga flux condition, as shown in RHEED pattern A*. With the increase in Ga flux, the growth mechanism can transfer to 2D mode and the optical properties of GaN films can be enhanced. In summary, the quality of the 2D MoS_2_ layer still plays an important role in the van der Waals epitaxy of GaN films [22].

### 3.9. EDS

**Figure 9:**
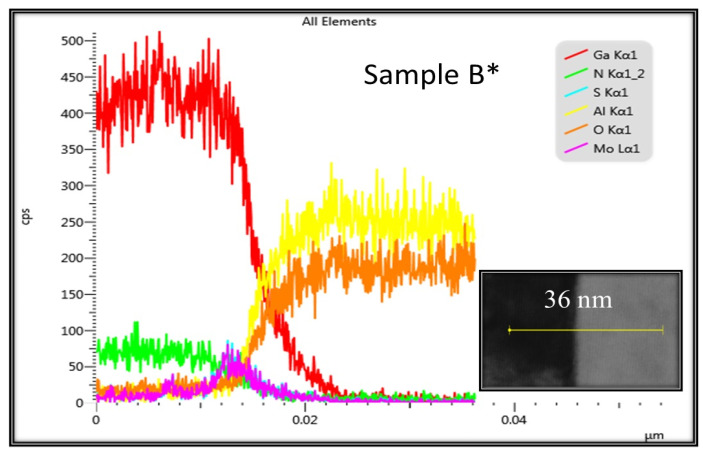
EDS spectra of GaN/MoS_2_/sapphire for samples B*.

Finally, EDS measurements were conducted to analyze the chemical composition of the heterostructure of GaN/MoS_2_ on sapphire. Figure 9 displays six elements in the line scan of 36 nm on sample B*. The red and green curves show Ga and N signals from GaN films, the yellow and orange curves show the elements of Al and O from the substrate sapphire, and the purple and blue curves show Mo and S elements from the 2D MoS_2_ layer at the interface between GaN and sapphire. The results of EDS spectra also confirm the growth of GaN films on 2D MoS_2_ layers by MBE.

## 4. Conclusions

In this paper, single-crystal and wafer-scale GaN thin films have been successfully grown on 2D CVD MoS_2_ layers using the low-temperature PA-MBE technique. GaN films grown at different K-cell temperatures of 825, 875, and 925 °C were characterized using RHEED, FE-SEM, AFM, XPS, Raman, PL, HR-XRD, TEM, and EDS. This investigation evidences the van der Waals epitaxy of GaN films on the 2D MoS_2_ layer and the integration of 3D GaN and 2D MoS_2_ heterostructures. In the epitaxial growth, the growth mechanism changes from 3D to 2D mode with the increase in Ga atom flux provided by the higher temperature of the Ga K-cell. The higher Ga flux leads to the formation of hexagonal crystals with multilevel steps on the surface. The highest percentage of GaN bonding can be obtained for the sample with a K-cell temperature of 925 °C. The crystalline quality and growth rate of GaN films can be enhanced by an increase in the Ga flux during MBE growth. Optical properties in the GaN films are also related to the quality of the crystal structure for GaN films. The adequacy of Ga flux not only facilitates an increase in the crystalline structure but also a decrease in the defect state. Additionally, the van der Waals bonding provided by 2D MoS_2_ layers helps minimize lattice distortion at the interface between GaN and MoS_2_, thereby reducing strain and defects in the GaN films. Its bonding facilitates the relaxation of strain and the incorporation of Ga and N atoms into the GaN lattice structure with minimal disruption. The heterostructures of GaN/MoS_2_ using MBE could be employed for applications in novel electronic and optoelectronic devices. To further advance the research in this field, higher crystal quality and smoother surfaces of the 2D layer will be necessary to reduce the dislocation density in the epitaxial GaN films and enhance the optical properties of GaN-based devices fabricated on 2D MoS_2_ layers.

## Figures and Tables

**Table 1:** Experimental parameters of MBE at different Ga K-cell temperatures.

Sample	A*	B*	C*
Substrate	MoS_2_/sapphire		
Thermal cleaning	600 °C for 30 min		
Pre-nitridation	600 °C for 10 min		
Gallium K-cell temperatures	825 °C	875 °C	925 °C
Growth temperature	750 °C for 3 hr		
Nitrogen plasma	500 W and 0.8 sccm		

**Table 2:** Peak positions and bonding percentages of the fitting of XPS spectra for three GaN films GaN/2D MoS_2_/sapphire.

Sample	A*	B*	C*
Ga-N position (eV)	20.13	19.78	19.18
Ga-Ga position (eV)	17.8	17.9	17.4
Ga-N (%)	90.9	90.6	98.4
Ga-Ga (%)	9.1	9.4	1.6

## Data Availability

The data presented in this study are available upon request to the corresponding authors.
